# Apical Secretion of FSTL1 in the Respiratory Epithelium for Normal Lung Development

**DOI:** 10.1371/journal.pone.0158385

**Published:** 2016-06-29

**Authors:** Xiaohe Li, Yinshan Fang, Xue Li, Jiurong Liang, Dianhua Jiang, Yan Geng, Wen Ning

**Affiliations:** 1 State Key Laboratory of Medicinal Chemical Biology, College of Life Sciences, Nankai University, Tianjin, 300071, China; 2 Cedars-Sinai Medical Center, Department of Medicine, Los Angeles, CA, 90048, United States of America; 3 School of Pharmaceutical Science, Jiangnan University, Wuxi, Jiangsu, 214122, China; Children's Hospital Los Angeles, UNITED STATES

## Abstract

Follistatin-like 1 (FSTL1) is a secreted bone morphogenetic protein (BMP) antagonist, and it plays a crucial role in normal lung development. Deletion of *Fstl1* leads to postnatal death in mice due to respiratory failure. To further explore the role of FSTL1 in mouse lung development, we created a transgene *SFTPC-Fstl1* allele mouse displaying significant epithelial overexpression of *Fstl1* in all stages of lung development. However, epithelial overexpression of *Fstl1* did not alter lung morphogenesis, epithelial differentiation and lung function. Moreover, we found that FSTL1 function was blocked by the epithelial polarization, which was reflected by the remarkable apical secretion of FSTL1 and the basolateral BMP signaling. Taken together, this study demonstrates that tightly spatial interaction of FSTL1 and BMP signaling plays an essential role in lung development.

## Introduction

The lung is optimized for oxygen supply of organism. To achieve this goal, the lung consists of two intertwined and precisely branched tree-like tubular systems to conduct air and blood supply. In the mouse embryo, lung development can be divided into embryonic (E9-E11.5), pseudoglandular (E11.5-E16.5), canalicular (E16.5-E17.5), terminal sac (E17.5-P5) and alveolar stages (P5-P30) [1–3]. Epithelial-mesenchymal interactions play an essential role in the complicated process of lung development, and such interactions are regulated by a variety of biochemical factors localized in the epithelium or the mesenchyme or both with different patterns of expression [2,3]. The extracellular molecules, including bone morphogenetic proteins (BMPs), TGF-βs, Wnts and FGFs, bind specific receptors and eventually activate combinations of transcription factors, thus guiding cellular proliferation and differentiation [1].

BMP4 signaling is crucial for lung development and its activity is tightly regulated by a family of secreted BMP antagonists. Changes of either BMP or BMP-antagonist expression can break the exquisite control system for lung development, resulting in deformities [4,5]. For example, disruption of BMP4 signaling in lungs of *SP-C-dnAlk6*, *SP-C-Xnoggin* or *SP-C-Gremlin* transgenic mice abrogates the proximal-distal patterning in the lung, where differentiation of distal epithelium is inhibited while proximal differentiation is promoted [5,6]. Mice overexpressing BMP4 or mice deficient for *Noggin* or *Gremlin*, show less extensive branching and decreased epithelial differentiation [4]. However, the precise mechanisms of BMPs and BMP-antagonists in lung development remain largely unclear.

Follistatin-like 1 (FSTL1), initially discovered as a TGF-β1-induced gene [7], encodes an extracellular glycoprotein, of which amino acid sequence is highly conserved among species [8–10]. It is a member of the secreted protein acidic and rich in cysteine (SPARC) family and follistatin family, based on the presence of a follistatin domain [7]. However, unlike other members of the SPARC family, its calcium-binding domain does not bind calcium [11]. Moreover, unlike follistatin and other follistatin-like proteins, FSTL1 does not bind activin [12]. The lack of conservation of important functional features common to several other family members indicates that FSTL1 is unique and has been evolved to acquire distinct properties [11]. Its functions and mechanisms have not been fully elucidated. Recently, we and other groups have identified the regulatory functions of FSTL1 in mouse organogenesis [13], including lung [12,14]. We have previously generated *Fstl1*-deficient mice and observed the postnatal lethality as a result of respiratory failure. Our data have shown that FSTL1 plays an important role in the differentiation/maturation of alveolar epithelial cells (AECs) during lung development via negatively regulating BMP4 signaling [12].

FSTL1 protein expression is dynamic during lung development, with widespread expression at the early stages, and then it becomes largely limited to mesenchymal tissues at the late stages [14–17], suggesting that the spatial interaction BMP4-FSTL1 plays a role in lung development. In this study, we further investigated the role of *Fstl1* in lung development by overexpressing it in the distal lung epithelium under the control of a Surfactant Protein-C (*SFTPC*) promoter [18]. We found that, unlike other BMP antagonists, overexpression of *Fstl1* did not alter the lung morphogenesis of *SFTPC-Fstl1* transgenic mice. In addition, its overexpression did not rescue the lethal lung phenotype of *SFTPC-Fstl1;Fstl1*^−/−^ compound mice. This puzzling phenotype could be interpreted by that an apically secreted FSTL1 was sequestrated from the basolateral BMP4 signaling in polarized AECs.

## Materials and Methods

### Ethics statement

All mice were housed and bred in a specific pathogen-free facility at Nankai University. All animal experiments were approved by the Animal Care and Use Committee at Nankai University (permit Number: 20140008). Pregnant mouse was sacrificed by cervical dislocation 18.5 days post-coitus, the uterus was removed, and the embryos were isolated quickly. After decapitation of the embryos, the lungs were rapidly harvested and used for subsequent experiments. All efforts were made to minimize suffering.

### Generation of *SFTPC-Fstl1* transgenic mice

The coding sequence region of murine *Fstl1* was generated by PCR from its full-length cDNA by using the following primers: sense 5'-GTCGACTCCCAC CTTCGCCTCTAACT-3', antisense 5'-GTCGACGCTGCAGACTCTGTGTGTAC-3'. The resulting 921-bp PCR product [18] was cloned into the *Sal* I site of the human *SFTPC* 3.7-kb promoter transgenic construct to generate the *SFTPC/Fstl1* transgenic vector. The expression cassette, a 5.3-kb fragment of a *SFTPC* promoter, a *Fstl1* coding sequence and an SV40 t intron-poly A, was released by *Not* I and *Nde* I nuclease digestion and used for tra*nsgenic* *micro*-*in*jection (S1 Fig). The transgenic embryos were identified by PCR reaction of tail DNA by using the following primers: sense 5'-TTGATTATGATGGGCACTG-3', antisense 5'-GAGGGTTGAAGGATGGG T-3'.

### Generation of compound mice (*SFTPC-Fstl1; Fstl1*^−/−^)

*Fstl1*^+/−^ mice have been generated as previously described [12]. The *SFTPC-Fstl1* transgenic mice were crossed with *Fstl1*^+/−^ mice to generate *SFTPC-Fstl1;Fstl1*^+/−^ mice, and the *SFTPC-Fstl1;Fstl1*^+/−^ mice were then crossed with *Fstl1*^+/−^ to generate compound mice (*SFTPC-Fstl1;Fstl1*^−/−^). The compound mice were genotyped by PCR of tail DNA by using the primers for *SFTPC-Fstl1* identification as above and primers for *Fstl1*^−/−^ identification as described [12].

### Cell culture and transfection

Mouse lung epithelial (MLE-12) cells (ATCC, cat.# CRL-2110^™^, USA) were purchased from ATCC and maintained in DMEM-F12 supplemented with 10% fetal bovine serum (FBS). Primary AECs were isolated as previously described [19]. Briefly, whole lungs at E18.5 were dissected and then digested with 1% type IV collagenase (Worthington, USA) at 37°C for 30 min to yield single cells. The filtered cell suspension was plated in 60-mm dishes and incubated at 37°C in a humidified 5% CO_2_ incubator for 1 h. The supernatant containing epithelium cells was removed and centrifuged at 1,000 r/min at room temperature for 10 min. The cell pellet was resuspended in DMEM-F12 containing 10% FBS, and cells were seeded onto collagen I-coated dishes. In monolayer cultures experiments, AECs and MLE-12 cells were seeded in 6-well plates at the density of 1×10^5^ (low density, LD) or 1×10^6^ (high density, HD). Cells were cultured for 1 day, serum-starved for 24 h and then treated with 20 ng/mL BMP4 in the presence or absence of 100 ng/mL FSTL1 for another 30 min. For Transwell cultures, cells were seeded in Transwell inserts (0.4 μm pore size, Corning, USA) at a density of 5×10^5^ and cultured for 48 h. After serum-starving for 24 h, cells were stimulated with BMP4 in the presence or absence of FSTL1 from either the apical or basal side. To overexpress FSTL1 in MLE-12, cells were plated in Transwell plates and transfected with 2 μg of *pc-Fstl1* using X-tremeGENE HP DNA Transfection Reagent (Roche). For transfection of E18.5 AECs, newly isolated cells were resuspended in 500 μL electroporation buffer, and then 10 μg BMPRII-FLAG plasmid was added. Subsequently, cells were transferred into a pre-chilled cuvette (inter-electrode distance of 2 mm). Electroporation was performed at 248 V (time constant: 5.8 ms) in a Bio-Rad Xcell Gene Pulser.

### Histology, immunohistochemistry and immunofluorescence

Whole embryos or lungs were fixed in 4% paraformaldehyde in PBS at 4°C and embedded in paraffin. Sections (5 μm) were mounted on slides, followed by deparaffinization. The sections were hydrated, heated in 10 mM citrate buffer (pH 6.0), treated with 3% H_2_O_2_ in PBS for 10 min and blocked with 5% normal goat serum. The sections were then incubated with primary antibodies, including anti-FSTL1 (Proteintech, China), anti-SCGB1A1 and anti-T1α antibodies (Santa Cruz, USA), at 4°C overnight. After extensive wash, samples were incubated with HRP-Polymer-goat anti-mouse/rabbit IgG (Fuzhou Maixin Biotech, China) for 15 min. Finally, the sections were developed with diaminobenzidine (DAB) (Fuzhou Maixin Biotech, China). For immunofluorescence analysis, the lung sections were incubated with primary antibodies, including anti-FSTL1 (Proteintech, China), anti-pro-SP-C (Abcam, UK) and anti-Endomucin (eBioscience, USA). Cells were fixed by 4% paraformaldehyde for 20 min, washed with PBS, permeabilized with 0.2% Triton X-100 in PBS, blocked with 5% BSA-PBS and incubated with anti-FLAG antibody (Sigma). After washed with PBS, donkey anti-rabbit Alexa Flour 555 or donkey anti-mouse Alexa Flour 488 secondary antibodies (Jackson ImmunoResearch, USA) were used for immunofluorescent visualization. Vectashield with DAPI was used as a mounting medium. Confocal images were obtained with a Leica TCS SP5 confocal microscope.

### Quantitative RT-PCR analysis

Total RNA was extracted with TRIzol (Invitrogen, USA). Isolated RNA was purified with the RNeasy Mini Kit (Qiagen, Germany) and the DNA-free kit (Ambion, USA). Subsequently, quantitative qRT-PCR was performed by using FastStart Universal SYBR Green Master mix (Roche) according to the manufacturer’s instructions. Gene expressions were determined relative to the endogenous reference gene (mouse *β-actin*) using the comparative CT method as previously described [20]. The sequences of qRT-PCR primers are listed in S1 Table.

### Mouse blood collection from orbital sinus

Blood samples were collected as previously described [21]. Briefly, mice were anesthetized, and then a microhematocrit tube was introduced to the canthus of the orbit. The microhematocrit tube was slightly advanced and rotated to let the blood flow into the tube. Samples were statically kept at room temperature for 1 h, and then centrifuged at 4°C. The supernatant was transferred to a clean tube, and the procedure was repeated one more time.

### Bronchoalveolar lavage (BAL)

BAL fluid was collected as previously described [22]. Briefly, mice were anesthetized, and the trachea was cannulated and lavaged four times with 1 mL sterile PBS. The BAL fluid was centrifuged at 4°C and the supernatant was transferred into a clean tube for protein extraction. The supernatant was precipitated with 100% Trichloroacetic Acid (TCA) and washed with acetone for two times. Extracted protein samples were stored at -80°C prior to further analysis.

### Protein extraction and western blotting

Mouse lung tissue was homogenized, and western blotting was performed as previously described [20]. The primary antibodies used were anti-FSTL1 (R&D Systems), phospho-Smad1/5/8, Smad5 (Cell Signaling Technology) and β-actin (Sigma) antibodies. Protein signals were detected using the enhanced chemiluminescence (ECL) kit (Pierce Biotechnology, USA). All experiments were repeated in triplicate.

### Statistical analysis

Data were expressed as the mean ± SEM. Differences between experimental and control groups were assessed by using two-sided Student t test. For multivariate data analysis, the general linear model (2-way ANOVA) with adjusted least-squares means was used to test the adjusted mean difference between groups. *P* < 0.05 was considered statistically significant.

## Results

### Generation of *SFTPC-Fstl1* transgenic mice

**Fig 1:**
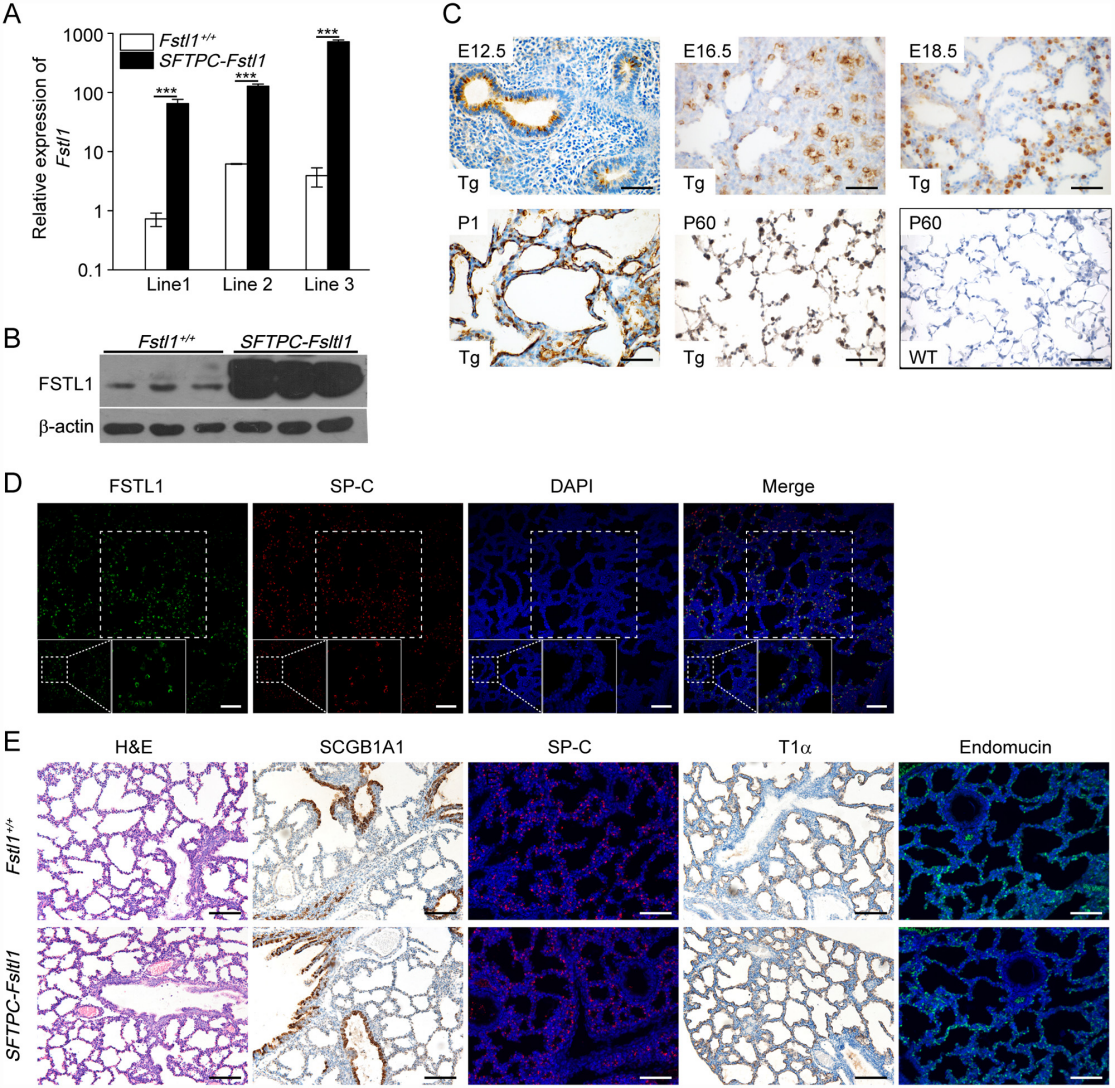
Characterization of *SFTPC-Fstl1* transgenic mouse lungs. (**A**) qRT-PCR analysis of *Fstl1* mRNA expression in E18.5 lungs from three lines of transgenic mice. n = 3. ***, *P* < 0.001. Data represent the mean ± SEM in triplicates. (**B**) Western blotting analysis of FSTL1 expression in E18.5 lungs. β-actin was used as a loading control. (**C**) Immunohistochemical staining for epithelial expression of FSTL1 in transgenic (Tg) lungs from E12.5 to P60. P60 WT staining was used as control. Scale bars, 50 μm. (**D**) Co-localization of FSTL1 and SP-C in distal region of E18.5 transgenic lungs. Scale bars, 25 μm. (**E**) Expression of differentiation markers for lung epithelial cells and microvascular network development in E18.5 lungs. Scale bars, 100 μm.

We previously showed that the loss of *Fstl1* leads to an impaired differentiation and maturation of distal AECs at saccular stage. We wondered whether epithelial expression of *Fstl1* could rescue the phenotype, since *Fstl1* is widely expressed by many tissue types including epithelium during early lung development [15]. To this end, we used the human *SFTPC* promoter [18] to drive the expression of mouse *Fstl1* (S1 Fig). Previous study has shown that the 3.7 kb promoter can specifically target the expression of many different transgenes in the lung epithelium with higher expression in the distal region [18]. Five female founders were obtained, and three of them passed the transgene *SFTPC-Fstl1* to their offsprings (S2 Fig). The *Fstl1* mRNA expression of above-mentioned mice was much higher than that of control littermates (Fig 1A). Among the three, the second line of the transgenic mouse (*SFTPC-Fstl1*) was back-crossed onto C57BL/6J background for at least 12 generations for further analysis.

Fig 1B shows that the transgenic mice had increased FSTL1 protein expression in the lung at E18.5. Immunohistochemical staining using an anti-FSTL1 antibody revealed the expression of FSTL1 protein in lung epithelium as early as E12.5 to adulthood (Fig 1C). Double immunofluorescence staining for FSTL1 and SP-C (a marker for distal epithelium) further confirmed that the FSTL1 expression was co-localized with SP-C-positive lung epithelium (Fig 1D). This expression pattern of transgene was well correlated with the reported pattern of *SFTPC* promoter activity in affected lungs [23].

### Normal lung morphology and differentiation in transgenic mice

All transgenic mice were viable and fertile, and they did not display any gross abnormalities. The transgene was transmitted in a Mendelian fashion. Fig 1E exhibits that both transgenic and wild-type (WT) lungs (E18.5) had many small distal saccules with thin septa and showed normal saccular expansion.

To assess whether *Fstl1* overexpression affected epithelial differentiation, we determined expression patterns of SCGB1A1 (a marker for conducting airway epithelial cells), SP-C, and T1α (a marker for type I AECs) by immunostaining. As shown in Fig 1E, sites and levels of SCGB1A1, SP-C and T1α remained unchanged in the respiratory epithelium of E18.5 transgenic mice as compared with their WT controls. Sections of E18.5 lungs of transgenic mice were also stained for Endomucin, a membrane glycoprotein marker expressed in endothelial cells, revealing normal number, size and location of capillaries (Fig 1E). Therefore, overexpression of *Fstl1* in the distal lung epithelium did not affect lung epithelial differentiation, microvascular network development and lung morphogenesis.

### Overexpression of *Fstl1* in lung epithelial cells fails to rescue the atelectasis phenotype of *Fstl1*^−/−^ mice

**Fig 2:**
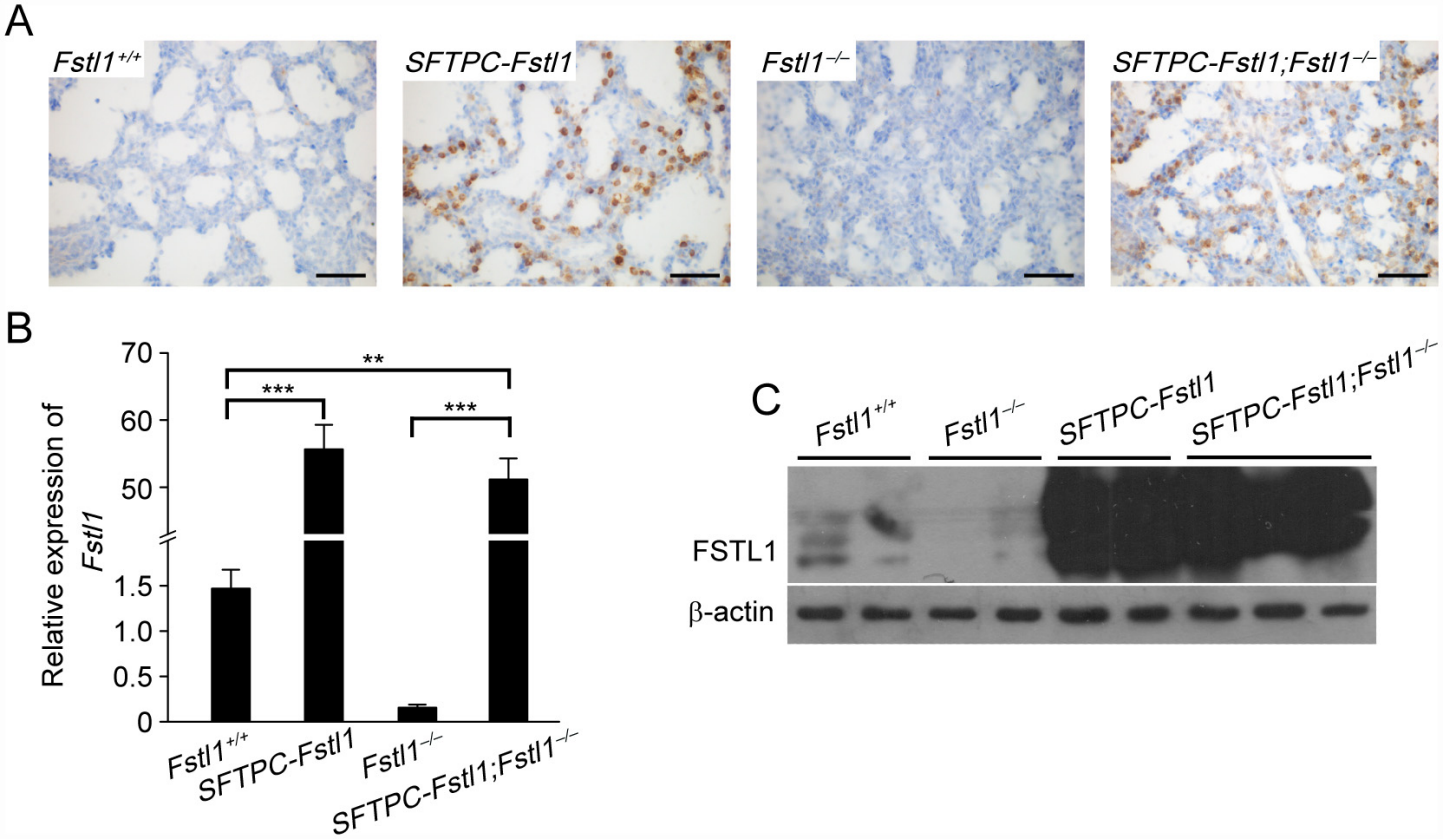
Characterization of *SFTPC-Fstl1*;*Fstl1*^−/−^ compound mouse lungs. (**A**) Immunohistochemical staining for FSTL1 expression in E18.5 lungs from different mutant mice. Scale bars, 50 μm. (**B**) qRT-PCR analysis of *Fstl1* mRNA expression in E18.5 lungs from different mutant mice. **, *P* < 0.01; ***, *P* < 0.001. Data represent the mean ± SEM in triplicates. (**C**) Western blotting analysis of FSTL1 expression in E18.5 lungs from different mutant mice. β-actin was used as a loading control.

To determine whether *Fstl1* overexpression in SP-C-expressing cells could rescue the atelectasis phenotype of *Fstl1*^−/−^ mice, *SFTPC-Fstl1* mice were crossed with *Fstl1*^+/−^ mice to generate the compound mice (*SFTPC-Fstl1; Fstl1*^−/−^) overexpressing *Fstl1* in the distal lung epithelium. Immunohistochemistry analysis confirmed the increased FSTL1 expression in lung epithelium in the compound mice (Fig 2A). Moreover, qRT-PCR and western blotting analyses showed that Fstl1 mRNA and protein expressions in E18.5 lung tissues of the compound mice were significantly higher levels than those of their WT or *Fstl1*^−/−^ littermates, while their expressions were comparable to those of their transgenic litters (Fig 2B and 2C).

**Fig 3:**
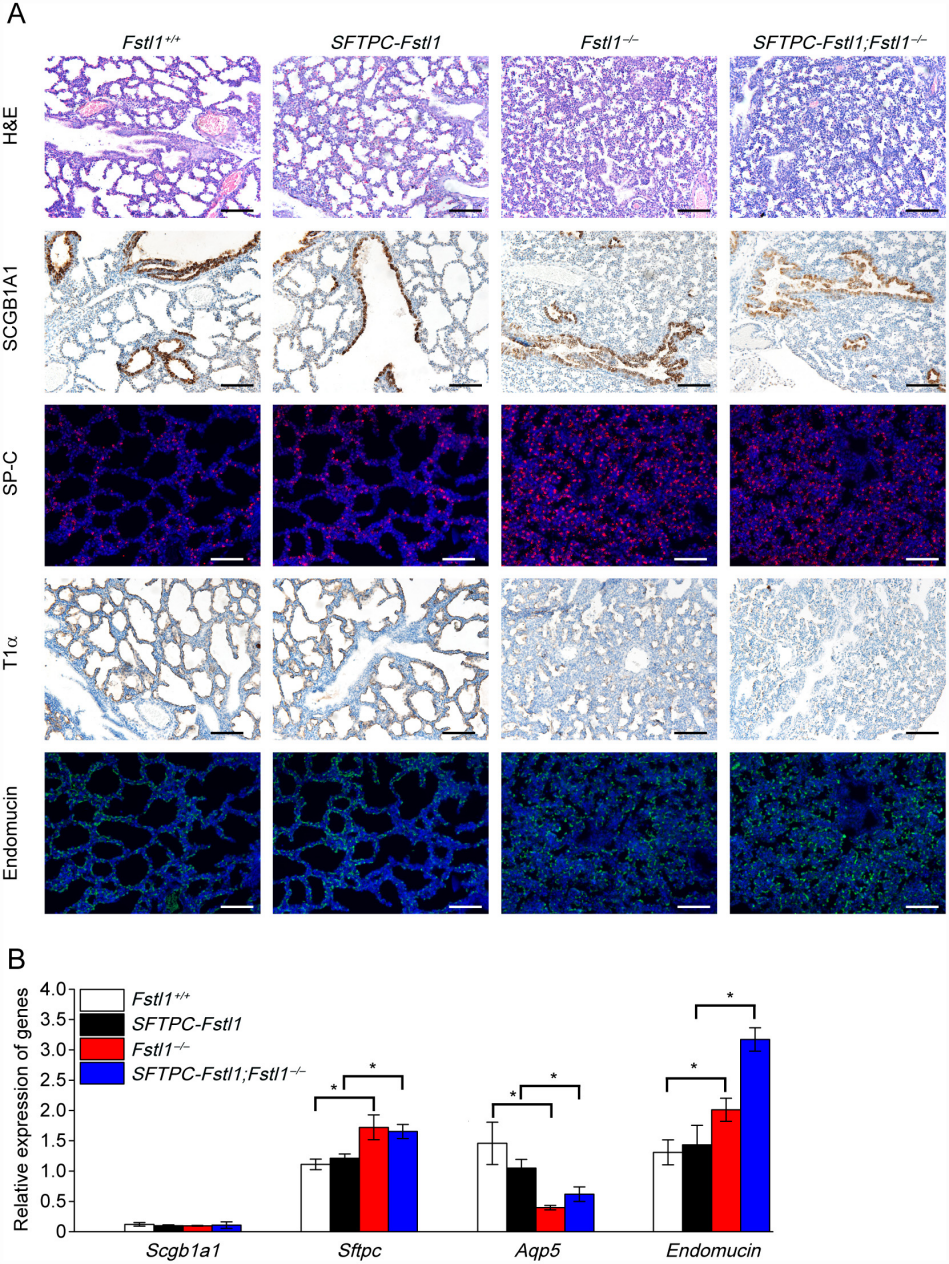
Overexpression of *Fstl1* in lung epithelium fails to rescue the atelectasis phenotype of *Fstl1*^−/−^ mice. (**A**) Expression of differentiation markers for lung epithelial cells and microvascular network development in E18.5 lungs from different mutant mice. Scale bars, 100 μm. (**B**) Relative expression levels of differentiation markers in E18.5 lungs from different mutant mice as determined by qRT-PCR. Data represent the mean ± SEM in triplicates. *, *P* < 0.05.

Surprisingly, epithelial overexpression of *Fstl1* did not rescue the lethal phenotype of *Fstl1*^−/−^ mice. Both *Fstl1*^−/−^ and compound litters died a few hours after birth. Histological examination revealed similar condensed appearance, as indicated by reduction in air sac spaces and thickened hypercellular intersaccular septa (E18.5) in lungs of *Fstl1*^−/−^ and compound litters (Fig 3A). By contrast, WT and transgenic mouse lungs displayed many small distal saccules with thin septa and showed normal saccular expansion (Figs 1E and 3A). Therefore, *Fstl1* overexpression in the distal lung epithelium failed to ameliorate or even rescue the lethal phenotype in *Fstl1*^−/−^ mice.

To test whether *Fstl1* overexpression effected lung epithelial differentiation in compound mice, the sections of E18.5 lungs were immunostained for markers for proximal and distal epithelium (Fig 3A). No significant differences were observed between the *Fstl1*^−/−^ and compound mice. Both strains displayed a comparable staining of SCGB1A1, an increased staining of SP-C and a significantly decreased staining of T1α compared with WT and transgenic littermates. This finding was also consistent with the results at the mRNA level, showing increased expression of *Sftpc* and decreased expression of *Aqp5* in *Fstl1*^−/−^ and compound mice compared with their WT and transgenic littermates (Fig 3B). These data suggested that the ectopic expression of *Fstl1* in lung epithelial cells did not rescue the impaired distal epithelial differentiation of *Fstl1*^−/−^ mice.

In the present study, we also assessed the distribution of blood capillaries in E18.5 lungs by immunostaining. Fig 3A shows that lungs of *Fstl1*^−/−^ and compound mice exhibited increased Endomucin staining compared with WT and transgenic littermates. In addition, qRT-PCR analyses confirmed the above observation (Fig 3B). These data suggested that the ectopic expression of *Fstl1* in the distal lung epithelium did not modify the abnormal lung epithelial differentiation and alveolar capillary network formation.

### Overexpression of *Fstl1* in distal lung epithelial cells does not affect BMP4/Smad signaling in transgenic mice

**Fig 4:**
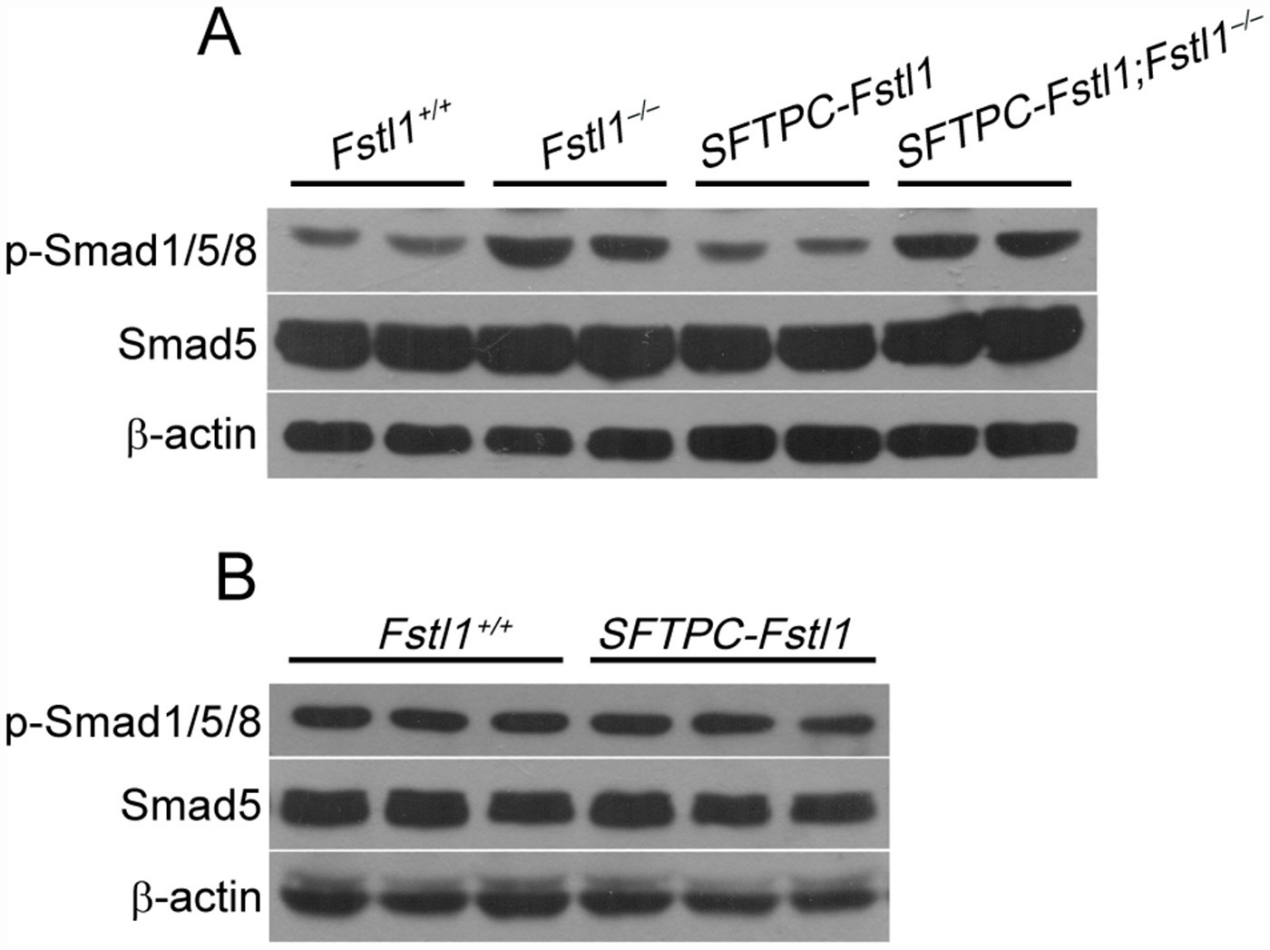
FSTL1-regulated BMP4 signaling in WT, *SFTPC-Fstl1* transgenic and compound mice. (**A**) Western blotting analysis of phosphorylated Smad1/5/8 in lungs from E18.5 WT (*Fstl1*^*+/+*^), *Fstl1*^−/−^, *SFTPC-Fstl1* and compound mice. (**B**) Western blotting analysis of phosphorylated Smad1/5/8 in lung tissues from P60 *SFTPC-Fstl1* transgenic mice and their WT littermates.

Since FSTL1 plays an important role in antagonizing BMP4 signaling in lung development [12], we expected that *Fstl1* overexpression would affect transgenic lungs with suppressed BMP4 signaling. Unfortunately, similar phosphorylation levels of Smad1/5/8 in E18.5 (Fig 4A) and P60 (Fig 4B) lungs were observed between *Fstl1* transgenic mice and their WT littermates. Remarkably, *Fstl1* overexpression did not reduce the increased phosphor-Smad1/5/8 levels in lungs of compound mice either (Fig 4A). Our data indicated that ectopic expression of *Fstl1* in lung epithelial cells did not affect BMP4/Smad signaling in lung epithelial cells. This finding seems inconsistent with our previous report [12] and argues the role of FSTL1 as a simple BMP antagonist molecule.

### Apical secretion of FSTL1

**Fig 5:**
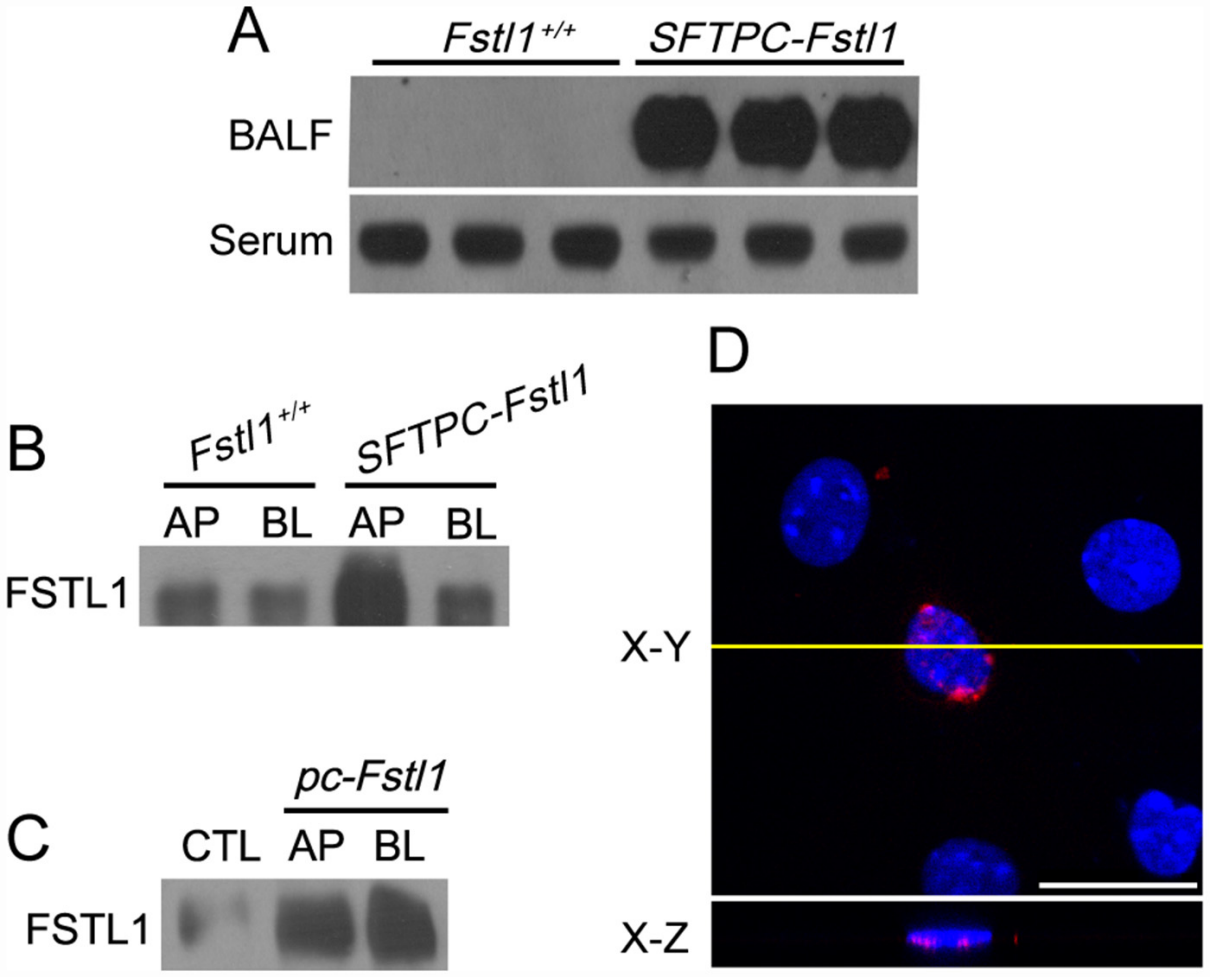
Apical secretion of FSTL1 and basolateral location of BMPRII in polarized lung epithelial cells. (**A**) FSTL1 levels in BAL and serum from adult *SFTPC-Fstl1* mice (P60) as determined by western blotting analysis. (**B-C**) Primary AECs **(B)** isolated from E18.5 *SFTPC-Fstl1* transgenic mice and MLE-12 cells **(C)** transfected with *pcDNA3*.*1-Fstl1* (*pc-Fstl1*). FSTL1 level in conditioned medium from the apical (AP) or basolateral (BL) side was determined by western blotting. (**D**) Primary mouse AECs from E18.5 lungs were stained with an anti-FLAG antibody (Red). X-Y (horizontal) and X-Z (vertical) sections are shown in the top and bottom panels, respectively. The yellow line shows the Y position of X-Z section. Scale bars, 7.5 μm.

FSTL1 is a secreted glycoprotein and can be detected in the peripheral blood [7,11,24,25]. Fig 5A shows that a large amount of FSTL1 was detected in BAL fluid instead of serum of adult *SFTPC-Fstl1* transgenic mice, indicating that the ineffective suppression on BMP signaling in transgenic mice was associated with an apical secretion of FSTL1 from epithelial cells. We next examined the apical secretion of FSTL1 in epithelial cells from transgenic mice. Freshly isolated AECs from E18.5 WT or transgenic mice were seeded onto Transwell plates and allowed to form polarized monolayers. Conditioned medium was collected from both sides, and FSTL1 levels were determined by western blotting analysis. Fig 5B shows that secretion of FSTL1 was more predominant from apical surface of AECs from both WT and transgenic mice. On the other hand, *Fstl1* overexpression in non-polarized MLE-12 cells showed similar levels of FSTL1 in conditioned medium from both sides (Fig 5C).

Considering a recent report of a basolateral BMP signaling in canine kidney polarized epithelial cells (MDCK) [26], we hypothesized that the apical secretion of FSTL1 was sequestrated from the basolateral BMP signaling in polarized AECs. To test this hypothesis, we first examined the localization of BMP receptors in mouse polarized AECs. AECs were freshly isolated from E18.5 mouse lung and transfected with a FLAG-tagged type II BMP receptor (BMPRII-FLAG). Immunofluorscence analyses using perpendicular sections shows that transient overexpression of BMPRII-FLAG was properly distributed to the basolateral surface of AECs (Fig 5D), demonstrating that BMPRII was basolaterally localized in polarized primary AECs.

### Apical secretion of FSTL1 is sequestrated from the basolateral BMP receptors in polarized lung epithelial cells

**Fig 6:**
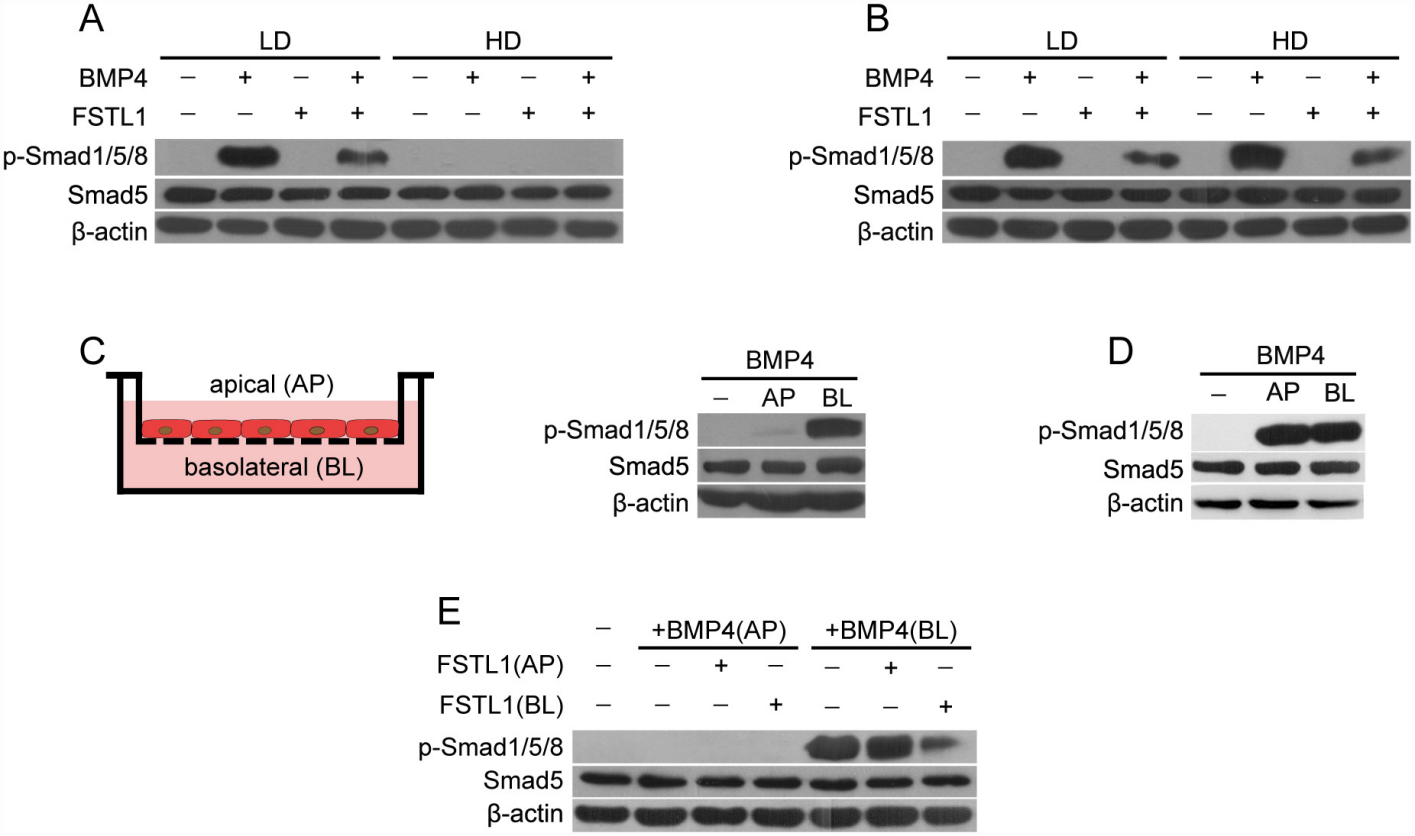
Apical secretion of FSTL1 has less effect on basolateral BMP signaling in polarized lung epithelial cells. (**A-B**) Western blotting analysis of p-Smad1/5/8 and Smad5 in newly isolated primary AECs from E18.5 lungs (**A**) or in MLE-12 cells **(B)**. Both cells were seeded at the density of 1 ×10^5^ (LD) or 1 ×10^6^ (HD) cells in 6-well plates and then treated with BMP4 (20 ng/mL) and FSTL1 (100 ng/mL) for 30 min. (**C-D**) Both primary AECs **(C)** and MLE-12 cells **(D)** were grown to confluence in Transwell plates and then treated with BMP4 (20 ng/mL) from the apical (AP) or basolateral (BL) side for 30 min. Phosphorylation of Smad1/5/8 induced by BMP4 was determined with western blotting. (**E**) Primary AECs from E18.5 lungs were grown to confluence in Transwell plates and then treated with BMP4 (20 ng/mL) and FSTL1 (100 ng/mL) from the apical (AP) or basolateral (BL) side for 30 min. Phosphorylation of Smad1/5/8 induced by BMP4 was determined with western blotting. **(A-E)** β-actin was used as a loading control.

We next examined the apical secretion of FSTL1 on BMP signaling. Newly isolated AECs were treated with BMP4 in monolayer culture under either sparse or confluent conditions. The phosphorylation of Smad1/5/8 was increased by BMP4 treatment under the sparse (LD) condition, whereas such an effect was not observed under the confluent (HD) condition (Fig 6A). As expected, FSTL1 could block BMP4-induced phosphorylation of Smad1/5/8 level only under the sparse condition (Fig 6A). On the other hand, BMP4 increased the phosphorylation of Smad1/5/8, and FSTL1 blocked BMP4-increased p-Smad1/5/8 levels in non-polarized MLE-12 under both culture conditions (Fig 6B).

Moreover, primary AECs were seeded onto Transwell plates, and polarized monolayers formed when cells reached confluence. BMP4 was added from either the apical or basolateral side, and phosphorylation of Smad1/5/8 was determined by western blotting. Fig 6C shows that the level of p-Smad1/5/8 was increased by BMP4 treatment from the basolateral side, whereas such an increase was not detected from the apical side. On the contrary, BMP4 treatment increased the phosphorylation of Smad1/5/8 in MLE-12 from both sides (Fig 6D). Therefore, our data demonstrated that BMP4 signals were only selectively transmitted from the basolateral surface in polarized AECs.

To investigate the role of FSTL1 on basolateral BMP4 signaling in polarized AECs, BMP4 or FSTL1 was administrated from either the apical or basolateral side, and phospharylation of Smad1/5/8 was determined by western blotting. As expected, BMP4-induced phospharylation of Smad1/5/8 could only be inhibited when both BMP4 and FSTL1 were administrated from the basolateral side (Fig 6E). Taken together, the apical secretion of FSTL1 in AECs was sequestrated from basolateral BMP4 signaling, resulting in ineffective suppression of BMP signaling and subsequent normal epithelial differentiation in transgenic mice.

## Discussion

As a secreted protein, FSTL1 has been implicated in many different biological processes, including development. The proposed mechanisms of most developmental defects observed in *Fstl1* KO mice are related to the disrupted BMP signaling [12,14,27]. These findings add FSTL1 to a group of secreted BMP antagonists, in which members are integral to the delicate control of BMP. Nevertheless, how FSTL1 affects BMP signaling remains largely unclear. In the present study, our data provided new insights into the understanding of the orientational nature of BMP signaling in polarized lung AECs and demonstrated that tightly spatial interaction of FSTL1 and BMP signaling plays an essential role in lung development. Epithelial overexpression of FSTL1 in *SFTPC-Fstl1* transgenic mice had less effect on epithelial differentiation and subsequent lung morphogenesis due to the sequestration of apically secreted FSTL1 from basolateral BMP4 signaling in polarized AECs.

We assessed the role of FSTL1 as a BMP antagonist in lung development. Our data showed that overexpression of *Fstl1* did not alter the lung morphogenesis of *SFTPC-Fstl1* transgenic mice. Moreover, it also did not rescue the impaired AEC differentiation of the *SFTPC-Fstl1;Fstl1*^−/−^ compound mice. Consistently, overexpression of *Fstl1* had less effect on BMP/Smad1/5/8 signaling activity in lungs of either transgenic or compound mice. This finding was similar to previous reports that no developmental defects have been described in zebrafish [28], frogs [10], or mice [29] upon overexpression of FSTL1. These puzzling facts seemed unlikely if FSTL1 is a "simple" BMP inhibitor molecule [13].

The lack of any obvious developmental phenotype in lungs of *SFTPC-Fstl1* transgenic mice could be associated with the orientational nature of BMP signaling in polarized epithelial cells. BMP4 has been implicated in the regulation of morphologically correct development of lung, specifically distal epithelial differentiation [4,5]. Its activated canonical signaling has been confirmed in a substantial portion of AECs using a *BRE-eGFP* reporter mouse line [30]. A previous study has shown that addition of BMP4 to E11.5 lungs grown in organ culture leads to a stimulation of branching and an increased cell proliferation, whereas direct administration of BMP4 into the lumen of lungs in organ culture has no effect on proliferation or branching [31]. Although a basolateral BMP signaling in canine kidney polarized epithelial cells has been reported [26], no evidence in lung epithelial cells has been proposed. In the present study, we showed that BMP4 transmitted its signals from the basolateral surface in polarized AECs. In newly isolated primary AECs, we identified the basolateral localization of BMP receptors, and found that the access of apically delivered BMP4 to its receptors was deprived, while delivery of basolateral ligands remained entirely effective to induce BMP responses.

BMP antagonist FSTL1 is a secreted glycoprotein produced mainly by cells of mesenchymal origin. Adams, *et al*. [15] discovered that *Fstl1* is highly expressed in the mesenchyme surrounding the larger airway. Sylva *et al*. [14] generated a *Fstl1-GFP* reporter mouse line and showed that the expression of *Fstl1* is detected in mesenchyme surrounding airways as well as in endothelium of blood vessels. Conventional deletion of *Fstl1* in mice increases BMP/Smad1/5/8 activity, causes perinatal lethality with abnormal thickness of septa walls and interferes with distal differentiation of lung epithelial cells at saccular stage of lung development [12]. However, in the present study, apical secretion of FSTL1 in the lung epithelium had less effect on BMP4 signaling and subsequent development phenotypes of transgenic or compound mice, reflecting the sequestration of antagonist from BMP signaling. In vitro data confirmed that BMP4-induced phospharylation of Smad1/5/8 could only be inhibited when both FSTL1 and BMP4 were administrated from basolateral side of newly isolated primary AECs. These findings suggest that tightly spatial interaction of FSTL1 and BMP signaling plays an essential role in lung development.

Interactions between ligands and receptors are vital for lung development and homeostasis. Apically expressed ligand and basolateral receptor pairs can be exampled by a growth factor Neregulin and its receptors ErbBs [32]. Neregulin is apically expressed, while its receptors ErbB 2–4 are located at basolateral membrane of differentiated airway epithelial cells. The sequestration and segregation of receptor and ligand provide a mechanism that helps the cell to maintain its current state, such as differentiation, and may be also important for rapid restoration of tissue integrity after injury. In the case of FSTL1 and BMP4 receptors, FSTL1 may be able to act onto BMP4 receptors when epithelial cells are immature in the early stage of lung development or injured during chronic lung diseases. Once the cell is fully differentiated/polarized, the receptor-ligand pair is segregated to maintain epithelial polarity.

In summary, we provided models of spatial events regulating epithelial BMP4/Smad signaling during lung development. FSTL1 exerted its negative regulation on BMP signaling depending on the spatial access of FSTL1 to BMP and BMP receptors in a cell-type-specific manner. In polarized AECs, apically secreted FSTL1 was deprived of the access of basolateral location of BMP receptors, thus failing to interfere with BMP receptor complex formation. Our findings could partially explain the puzzling fact, raised by Sylva *et al*. [13], that no developmental defects have been described in zebrafish, frogs or mice upon overexpression of FSTL1. FSTL1 is unlike the other known extracellualr BMP antagonists, such as Noggin and Gremlin. However, our laboratory is actively pursuing insights into a possible regulatory role of FSTL1 in AEC differentiation, TGF-β1 and other signaling pathways during lung morphogenesis and lung homeostasis, as well as the interplay of FSTL1 with BMP4 and TGF-β1. This study and our continuing efforts will provide a better understanding of the mechanism coordinating the TGF-β superfamily during lung morphogenesis.

## Supporting Information

S1 Fig Schematic diagram of the transgenic DNA construct. Mouse *Fstl1* was driven by a 3.7-kb *SFTPC* promoter, followed by an SV40 small T intron and polyA tail.
(TIF)

S2 Fig Screening of transgenic founder lines by PCR using genomic DNA. The genomic DNA from mouse tail was used for PCR amplification. N: negative control; P: positive control. Amplicon size was 411 bp.
(JPG)

S1 Table Sequences of qRT-PCR primers. (DOCX)
