# Supplementary figures and images for: Apical Secretion of FSTL1 in the Respiratory Epithelium for Normal Lung Development
Source: PLoS One. 2016 Jun 29;11(6):e0158385. doi: 10.1371/journal.pone.0158385 (PMC4927184; doi:10.1371/journal.pone.0158385)

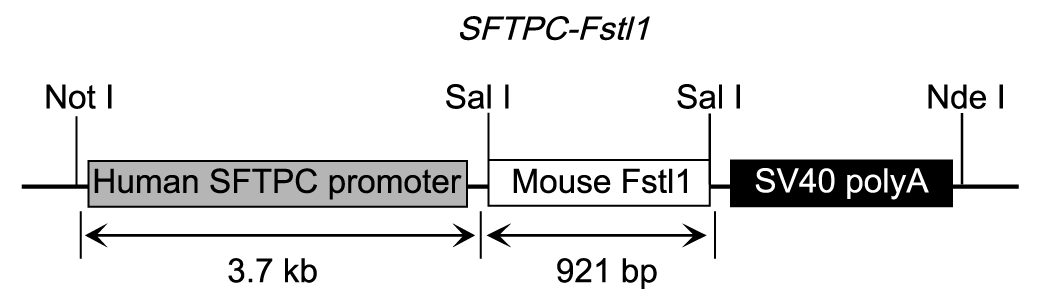

Supplement: S1 Fig — Mouse Fstl1 was driven by a 3.7-kb SFTPC promoter, followed by an SV40 small T intron and polyA tail. (TIF) [file pone.0158385.s001.tif]

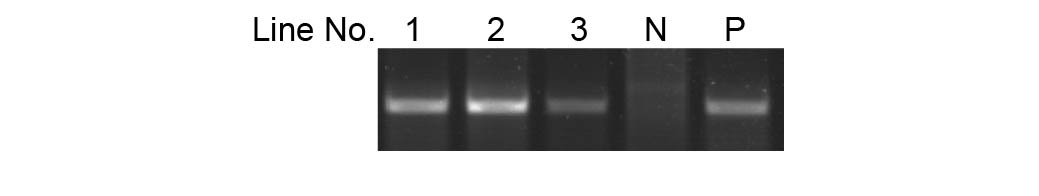

Supplement: S2 Fig — The genomic DNA from mouse tail was used for PCR amplification. N: negative control; P: positive control. Amplicon size was 411 bp. (JPG) [file pone.0158385.s002.jpg]
