# Supplementary material for: Apical Secretion of FSTL1 in the Respiratory Epithelium for Normal Lung Development
Source: PLoS One. 2016 Jun 29;11(6):e0158385. doi: 10.1371/journal.pone.0158385 (PMC4927184; doi:10.1371/journal.pone.0158385)
Supplement: S1 Table — (DOCX) [file pone.0158385.s003.docx]

**S1 Table. Sequences of qRT-PCR primers.**

| **Target** | **Forward** | **Reverse** |
| --- | --- | --- |
| *Sftpc* | GAAGATGGCTCCAGAGAGCATC | GGACTCGGAACCAGTATCATGC |
| *Aqp5* | GGTGGTCATGAATCGGTTCAGC | GTCCTCCTCTGGCTCATATGTG |
| *Scgb1a1* | CATGCTGTCCATCTGCTGC | CTCTTGTGGGAGGGTATCC |
| *Endomucin* | caactacggcatgttttcca | gaggaaccaacacaatttcca |
| *Fstl1* | TTATGATGGGCACTGCAAAGAA | ACTGCCTTTAGAGAACCAGCC |
| *β-actin* | AGGCCAACCGTGAAAAGATG | AGAGCATAGCCCTCGTAGATGG |
